# Adherence to optimal heart rate control in heart failure with reduced ejection fraction: insight from a survey of heart rate in heart failure in Sweden (HR-HF study)

**DOI:** 10.1007/s00392-017-1146-6

**Published:** 2017-08-09

**Authors:** M. Fu, U. Ahrenmark, S. Berglund, C. J. Lindholm, A. Lehto, A. Månsson Broberg, G. Tasevska-Dinevska, G. Wikstrom, A. Ågard, B. Andersson, All investigators of the HR-HF study, Michael Fu, Bert Andersson, Sven Eric Hagelind, Stefen Berglund, John-Erik Frisell, Bertil Borgencrantz, Agneta Månsson Broberg, Ulla Wedén, Carl Thorsén, Fredrik Kymle, Gordana Tasevska, Anders Kullberg, Ulf Ahremark, Lars Andersson, Anders Ågård, Anette Lehto, Niels Wagner, Gerhard Wikström, Magnus Ehrsson, Julio Loayza, Carl-Johan Lindholm, Erasmus Bachus

**Affiliations:** 1 Department of Molecular and Clinical Medicine, Institute of Medicine, Sahlgrenska Academy, University of Gothenburg, Göteborg, Sweden; 2 Department of Medicine, Hospital in Halmstad, Halmstad, Sweden; 3 Department of Medicine, Hospital in Falun, Falun, Sweden; 4 Capio City Clinic, Lund, Sweden; 5 Department of Medicine, Northern Älvsborg County Hospital, Trollhättan, Sweden; 6 Division of Cardiology, Department of Medicine, Huddinge, Karolinska Institutet, Karolinska University Hospital Stockholm, Stockholm, Sweden; 7 Department of Cardiology, Malmö University Hospital, University of Lund, Malmö, Sweden; 8 Department of Cardiology, Academic University Hospital, Uppsala University, Uppsala, Sweden; 9 Department of Medicine, Angered Hospital, Göteborg, Sweden; 10 Section of Cardiology, Department of Medicine, Sahlgrenska University Hospital/Östra Hospital, 416 50 Göteborg, Sweden

**Keywords:** Heart rate, Heart failure, Awareness, Adherence, Beta-blocker

## Abstract

**Introduction:**

Despite that heart rate (HR) control is one of the guideline-recommended treatment goals for heart failure (HF) patients, implementation has been painstakingly slow. Therefore, it would be important to identify patients who have not yet achieved their target heart rates and assess possible underlying reasons as to why the target rates are not met.

**Materials and methods:**

The survey of HR in patients with HF in Sweden (HR-HF survey) is an investigator-initiated, prospective, multicenter, observational longitudinal study designed to investigate the state of the art in the control of HR in HF and to explore potential underlying mechanisms for suboptimal HR control with focus on awareness of and adherence to guidelines for HR control among physicians who focus on the contributing role of beta-blockers (BBs).

**Results:**

In 734 HF patients the mean HR was 68 ± 12 beats per minute (bpm) (37.2% of the patients had a HR >70 bpm). Patients with HF with reduced ejection fraction (HFrEF) (*n* = 425) had the highest HR (70 ± 13 bpm, with 42% >70 bpm), followed by HF with preserved ejection fraction and HF with mid-range ejection fraction. Atrial fibrillation, irrespective of HF type, had higher HR than sinus rhythm. A similar pattern was observed with BB treatment. Moreover, non-achievement of the recommended target HR (<70 bpm) in HFrEF and sinus rhythm was unrelated to age, sex, cardiovascular risk factors, cardiovascular diseases, and comorbidities, but was related to EF and the clinical decision of the physician. Approximately 50% of the physicians considered a HR of >70 bpm optimal and an equal number considered a HR of >70 bpm too high, but without recommending further action. Furthermore, suboptimal HR control cannot be attributed to the use of BBs because there was neither a difference in use of BBs nor an interaction with BBs for HR >70 bpm compared with HR <70 bpm.

**Conclusion:**

Suboptimal control of HR was noted in HFrEF with sinus rhythm, which appeared to be attributable to physician decision making rather than to the use of BBs. Therefore, our results underline the need for greater attention to HR control in patients with HFrEF and sinus rhythm and thus a potential for improved HF care.

## Introduction

Available international guidelines for heart failure (HF) with reduced ejection fraction (HFrEF) recommend the following pharmacological therapies: angiotensin-converting enzyme inhibitors (ACEIs) or angiotensin receptor blockers (ARBs) if the patient is intolerant to ACEIs, beta-blockers (BBs), mineralocorticoid receptor antagonists (MRAs), ivabradine and sacubitril–valsartan [1–3]. Although the implementation of clinical guidelines generally takes time, we have witnessed a gradual improvement and increased adherence to treatment with ACEIs/ARBs, BBs, and MRAs across different countries in the past two decades [4–8]. For example, the prescription of BBs has increased in Europe from 37% in 2000 to 87–91% today [6–8]. However, for newer drugs, such as ivabradine, implementation has been slower. For instance, Dierckx et al. reported that of patients with HFrEF, 94% were treated with BBs and only 4% were taking ivabradine [9]. One possible reason is physician-related factors, such as lack of awareness of and/or adherence to optimal heart rate (HR) control as part of the treatment goal in HFrEF and sinus rhythm. Lack of adherence has previously been suggested as one contributing factor for suboptimal HF care [10–13]. Another reason is assumed to be due to differences in use of BBs between Sweden and other countries. BBs are frequently used in the treatment of HFrEF in Sweden and could, therefore, contribute to better HR control and hence decrease the indication for further HR reduction with ivabradine. At present, while prescriptions of BBs are largely similar between Sweden and rest of the world [7, 14], the mean doses of BBs were higher in Sweden than those in other countries [6–10, 14]. According to the Swedish Heart Failure Registry (SwedeHF, *n* = 69,527, mean age 75 years), 67% of the patients with HFrEF were treated with BBs at ≥50% of the target doses. Among those <65 years, 77% of male and 68% of female patients were at ≥50% of the target doses [14]. However, according to the QUALIFY global registry, only 52% of HFrEF patients (mean age 63 years) were treated with BBs in ≥50% of the target doses [9]. Therefore, lower use of ivabradine in Sweden was assumed to be related to the more effective use of BBs.

The survey of HR in patients with HF in Sweden (HR-HF) was an investigator-initiated, prospective, multicenter, observational longitudinal study designed to investigate the status of HR control in an outpatient cohort of stable patients with HFrEF compared with patients with HF and mid-range ejection fraction (HFmrEF) and HF with preserved ejection fraction (HFpEF) in both sinus rhythm and AF. Moreover, we explored underlying reasons to suboptimal HR control.

The main objective of the study was to assess awareness of an adherence to HR control among physicians, particularly as it contributed to the use of BBs (prescription and doses). We hypothesized that a substantial proportion of patients would have HRs above 70–75 bpm.

## Materials and methods

### Protocol of the HR-HF study

The HR-HF study was a prospective, multicenter, observational longitudinal survey of HF outpatients that included 734 patients in 27 centers in Sweden. These centers were hospital HF outpatient clinics with either dedicated HF nurse specialists or general practitioners. Eligible patients were those with established HF in an outpatient setting and considered on stable HF medication regimens.

The survey was carried out from 2014 to 2016 with a planned follow-up from 2017. The following variables were recorded as baseline data: demographics, diagnostic validation with left ventricular ejection fraction (LVEF), N-terminal pro-b-type natriuretic peptide (NT-pro-BNP) or B-type natriuretic peptide (BNP), hospitalizations due to HF in the past 2 years, cardiovascular risk factors, cardiovascular diseases, non-cardiovascular diseases, symptoms (breathlessness, tiredness and chest pain, Likert scale), blood pressure (sitting, standing, lying), HF and rhythm (by ECG), New York Heart Association (NYHA) functional class, ADL (activity of daily living), use of BBs (up-titration, ≥50% of the target dose, target dose or above target dose, reasons for not being on BB treatment, reasons for not achieving target dose, side effects), use of ACEIs/ARBs/MRAs (up-titration, dose, reasons for not on treatment, reasons for not achieving target dose, side effects), other pharmacologic treatments, cardiac resynchronization therapy (CRT) device, implantable cardioverter defibrillator (ICD) device, and physicians’ judgment regarding actual HR.

Different from most available HF registries [6–8, 14], the HR-HF survey focused on stable HF patients and only in outpatient settings with a special interest in HF control. Further, there was a dedicated focus on collecting information that might influence HF, for example, comorbidities and their gradings, symptoms and gradings, blood pressure, medications (prescriptions, dose, tolerability, side effects), and clinical judgment in relation to HF.

This study adhered to the guidelines available for human studies, including an approved ethical permit, which complies with the Helsinki Declaration and the International Ethical Guidelines for Good Clinical Practice. The study was approved by the Regional Ethical Review Board at the University of Gothenburg.

### Study population

Patients eligible for entry into the survey were outpatient adults (>18 years old) with a well-established diagnosis of HF based on the latest European Society of Cardiology guidelines [1, 3] and according to the responsible investigator’s clinical judgment; an abnormal echocardiography investigation that was congruent with the HF diagnosis; optimal treatment (physicians decision) and are, therefore, not planned for further up-titration; and a stable HF condition and plans for further outpatient follow-up. The LVEF cutoffs used to define HFrEF, HFmrEF, and HFpEF were <40, 40–49, and ≥50%, respectively. No exclusion criteria were applied, except for those who did not or could not provide informed consent.

### Baseline evaluation and data management

Data were collected centrally using a case report form that was sent to the data management center, where checks for completeness, internal consistency, and accuracy were run. Forty-nine patients were excluded from the database because of protocol deviations or incompleteness.

### Statistical analysis

For categorical variables, *n*(%) was presented. For continuous variables, mean (SD)/median (Min/Max/*n*) was presented. For comparison between the three EF groups, the Mantel–Haenszel Chi-square statistic was used for ordered categorical variables, the Chi-square test for non-ordered categorical variables, and the Jonckheere–Terpstra test for continuous variables. For comparison between groups in different HRs, Fisher’s exact test (lowest one-sided *p* value multiplied by 2) was used for dichotomous variables, the Mantel–Haenszel Chi-square test for ordered categorical variables, and the Mann–Whitney *U* test for continuous variables. For interaction and subgroup analyses in reaching a HR > 70 bpm, logistic regression was performed and odds ratios (ORs) with associated 95% confidence intervals (CIs) and *p* values are presented from these analyses.

All tests were two-tailed and *p* values <0.05 were considered significant. All analyses were performed using SAS software version 9.4 (Cary, NC, USA).

## Results

### Patient characteristics in the overall cohort

Patient demographics, cardiovascular risk factors, cardiovascular diseases, non-cardiovascular diseases, clinical status, medications, and clinical assessment are outlined in Tables 1 and 2. Briefly, despite that patients with HFrEF were more often male, had more ischemic heart disease, higher NT-pro-BNP, more ventricular extrasystolic couplets (VECs)/ventricular tachycardia (VT), lower blood pressure, and more left bundle branch block (LBBB), they had a similar number of non-cardiovascular co-morbidities compared with HFmrEF and HFpEF.

**Table 1:** Baseline data for demographics, risk factors, and medical histories

Variable	Total (*n* = 734)	HFrEF (*n* = 425)	HFmrEF (*n* = 187)	HFpEF (*n* = 122)	*p* value
Demographics					
Age (years)	69.1 (11.6) 70.6 (19.0; 95.3)	69.8 (11.2) 71.6 (19.0; 95.3)	67.8 (12.3) 69.8 (20.8; 89.8)	68.7 (11.8) 69.2 (30.0; 89.7)	0.11
Male	549 (74.8%)	337 (79.3%)	133 (71.1%)	79 (64.8%)	0.0004
Cardiovascular risk factors					
Hypertension	388 (52.9%)	213 (50.1%)	92 (49.2%)	83 (68.0%)	0.0033
BMI >30 kg/m^2^	209 (28.5%)	121 (28.5%)	48 (25.7%)	40 (32.8%)	0.57
Diabetes	181 (24.7%)	112 (26.4%)	33 (17.6%)	36 (29.5%)	0.88
Hypercholesterolemia	258 (35.3%)	164 (38.9%)	56 (29.9%)	38 (31.4%)	0.045
Stress	179 (24.5%)	98 (23.1%)	53 (28.5%)	28 (23.0%)	0.66
Cardiovascular diseases					
Ischemic heart disease	339 (46.2%)	218 (51.3%)	82 (43.9%)	39 (32.0%)	0.0001
Primary valvular disease	89 (12.1%)	46 (10.8%)	19 (10.2%)	24 (19.7%)	0.028
Cardiomyopathy	243 (33.1%)	152 (35.8%)	57 (30.5%)	34 (27.9%)	0.067
Chronic persistent atrial fibrillation	201 (27.4%)	120 (28.2%)	42 (22.5%)	39 (32.0%)	0.83
Paroxysmal atrial fibrillation	119 (16.2%)	68 (16.0%)	24 (12.8%)	27 (22.1%)	0.28
VES/VT	130 (17.7%)	84 (19.8%)	34 (18.2%)	12 (9.8%)	0.019
Non-cardiovascular diseases					
Mild/moderate pulmonary disease	70 (9.5%)	43 (10.1%)	15 (8.0%)	12 (9.8%)	0.73
Severe pulmonary disease	13 (1.8%)	7 (1.6%)	3 (1.6%)	3 (2.5%)	0.61
GFR <30 ml/min	34 (4.7%)	21 (5.0%)	8 (4.3%)	5 (4.1%)	
30–60 ml/min	257 (35.3%)	163 (38.6%)	51 (27.6%)	43 (35.5%)	
>60 ml/min	437 (60.0%)	238 (56.4%)	126 (68.1%)	73 (60.3%)	0.14
Stroke without sequelae	62 (8.4%)	33 (7.8%)	17 (9.1%)	12 (9.8%)	0.42
Stroke with sequelae	23 (3.1%)	15 (3.5%)	5 (2.7%)	3 (2.5%)	0.48
Hemoglobin (g/L) (cat.)					
<90	4 (0.6%)	2 (0.5%)	2 (1.2%)	0 (0.0%)	
90 to <110	29 (4.5%)	22 (6.0%)	3 (1.8%)	4 (3.6%)	
≥110	611 (94.9%)	342 (93.4%)	161 (97.0%)	108 (96.4%)	0.14
Depression	81 (11.0%)	47 (11.1%)	20 (10.7%)	14 (11.5%)	0.95
Impotence	140 (29.3%)	93 (32.0%)	35 (28.2%)	12 (19.0%)	0.046
Malignancy (active)	15 (2.0%)	9 (2.1%)	4 (2.1%)	2 (1.6%)	0.78
Malignancy (stable)	73 (9.9%)	45 (10.6%)	13 (7.0%)	15 (12.3%)	0.98
Malnutrition	24 (3.3%)	16 (3.8%)	5 (2.7%)	3 (2.5%)	0.40
Liver failure	6 (0.8%)	3 (0.7%)	1 (0.5%)	2 (1.6%)	0.42
Thyroid disease	60 (8.2%)	28 (6.6%)	17 (9.1%)	15 (12.3%)	0.037
Gout	97 (13.2%)	64 (15.1%)	15 (8.0%)	18 (14.8%)	0.39
Dementia	3 (0.4%)	2 (0.5%)	1 (0.5%)	0 (0.0%)	0.56
Other important non-cardiovascular disease	65 (8.9%)	32 (7.5%)	14 (7.5%)	19 (15.6%)	0.018

**Table 2:** Baseline data for clinical status, medication, and clinical assessment by physicians

Variable	Total (*n* = 734)	HFrEF (*n* = 425)	HFmrEF (*n* = 187)	HFpEF (*n* = 122)	*p* value
Clinical status					
LVEF (%)	36.9 (17.9) 35.0 (10.0; 401.0) *n* = 734	28.2 (6.8) 30.0 (10.0; 39.0) *n* = 425	43.0 (2.8) 42.5 (40.0; 49.0) *n* = 187	57.7 (31.7) 55.0 (50.0; 401.0) *n* = 122	<.0001
NT-pro-BNP (ng/L)	2810 (5044) 1251 (10; 70,000) *n* = 629	3255 (5345) 1559 (10; 70,000) *n* = 364	2021 (3936) 808 (37; 30,000) *n* = 156	2456 (5291) 706 (43; 35,000) *n* = 109	<.0001
Sitting systolic blood pressure (mmHg)	126.2 (58.2) 120.0 (54.0; 1500.0) *n* = 619	121.8 (17.8) 120.0 (54.0; 190.0) *n* = 341	126.1 (18.0) 126.0 (85.0; 180.0) *n* = 170	140.2 (133.4) 126.5 (85.0; 1500.0) *n* = 108	0.0010
Heart rate (bpm) by ECG	68.4 (12.4) 67.0 (34.0; 123.0) *n* = 734	69.8 (13.0) 68.0 (34.0; 123.0) *n* = 425	65.5 (10.7) 64.0 (43.0; 95.0) *n* = 187	68.1 (12.0) 66.0 (44.0; 103.0) *n* = 122	0.0062
<60 bpm	163 (22.2%)	81 (19.1%)	56 (29.9%)	26 (21.3%)	
60–70 bpm	298 (40.6%)	166 (39.1%)	77 (41.2%)	55 (45.1%)	
>70 bpm	273 (37.2%)	178 (41.9%)	54 (28.9%)	41 (33.6%)	0.019
LBBB	163 (22.2%)	111 (26.2%)	37 (19.8%)	15 (12.3%)	0.0007
Sinus rhythm (and not previously detected persistent or paroxysmal atrial fibrillation)	387 (52.8%)	216 (50.9%)	115 (61.5%)	56 (45.9%)	0.96
Atrial fibrillation (or previously detected persistent or paroxysmal)	322 (43.9%)	191 (45.0%)	66 (35.3%)	65 (53.3%)	0.51
Chamber pacing	149 (20.3%)	105 (24.8%)	26 (13.9%)	18 (14.8%)	0.0020
NYHA (cat.)					
I–II	538 (73.3%)	301 (70.8%)	150 (80.2%)	87 (71.3%)	
III–IV	196 (26.7%)	124 (29.2%)	37 (19.8%)	35 (28.7%)	0.37
Medication					
Beta-blockers	705 (96.0%)	411 (96.7%)	176 (94.1%)	118 (96.7%)	0.62
RAAS (ACEI/ARB)	707 (96.3%)	413 (97.2%)	180 (96.3%)	114 (93.4%)	0.065
MRA	407 (55.4%)	257 (60.5%)	93 (49.7%)	57 (46.7%)	0.0017
Loop diuretics	420 (57.2%)	267 (62.8%)	84 (44.9%)	69 (56.6%)	0.015
Digitalis	96 (13.1%)	61 (14.4%)	14 (7.5%)	21 (17.2%)	0.96
Statin	417 (56.8%)	260 (61.2%)	97 (51.9%)	60 (49.2%)	0.0062
Ivabradine/procoralan	21 (2.9%)	12 (2.8%)	8 (4.3%)	1 (0.8%)	0.50
Device treatments					
Conventional pacemaker	63 (8.6%)	29 (6.8%)	16 (8.6%)	18 (14.8%)	0.0091
CRT	106 (14.4%)	85 (20.0%)	15 (8.0%)	6 (4.9%)	<.0001
ICD	140 (19.1%)	110 (25.9%)	19 (10.2%)	11 (9.0%)	<.0001
Clinical assessment					
Physician considers patient having too low heart rate	21 (2.9%)	12 (2.8%)	6 (3.2%)	3 (2.5%)	0.92
Physician considers patient having optimal heart rate	568 (77.4%)	320 (75.3%)	152 (81.3%)	96 (78.7%)	0.22
Physician considers patient having too high heart rate	145 (19.8%)	93 (21.9%)	29 (15.5%)	23 (18.9%)	0.22

### Medications in the overall cohort

There were no differences in the use of BBs and ACEIs/ARBs between the groups of HF patients, regardless of EF, with 94–97% of the patients on treatment with BBs and 93–97% on treatment with ACEIs/ARBs (Table 2). However, in patients with HFrEF more patients were treated with MRAs, diuretics, statins, and therapy devices (CRT, ICD). In addition, patients with HFrEF were well treated with BBs (97%), ACEIs/ARBs (97%), MRAs (61%), CRT (20%), ICD (25) 9%, whereas only 2.8% had ivabradine.

Concerning doses of BBs, these were similar in HFrEF, HFmrEF, and HFpEF. Percentage of achieved target dose ≥50% was 79% for HFrEF, 75% for HFmrEF, and 85% for HFpEF. For reached target dose, it was 43% for HFrEF, 45% for HFmrEF, and 44% for HFpEF. Moreover, 6% (HFrEF), 5% HFmrEF, and 5% (HFpEF) of the patients had a dose above the target dose.

The main reasons why patients with HFrEF were not on treatment with BBs (3%) were low blood pressure (22.6%), bradycardia (15.9%), fatigue (9.6%), and dizziness (9.6%). Despite that, about 97% of the patients were on treatment with BBs (only 60.6% did not report side effects). The most frequently reported side effects were tiredness (20%), cold extremities (8.8%), impotence (8.3%), nightmares (3.2%), and depression (3.2%).

### Distribution of HR in the overall cohort

**Fig. 1:**
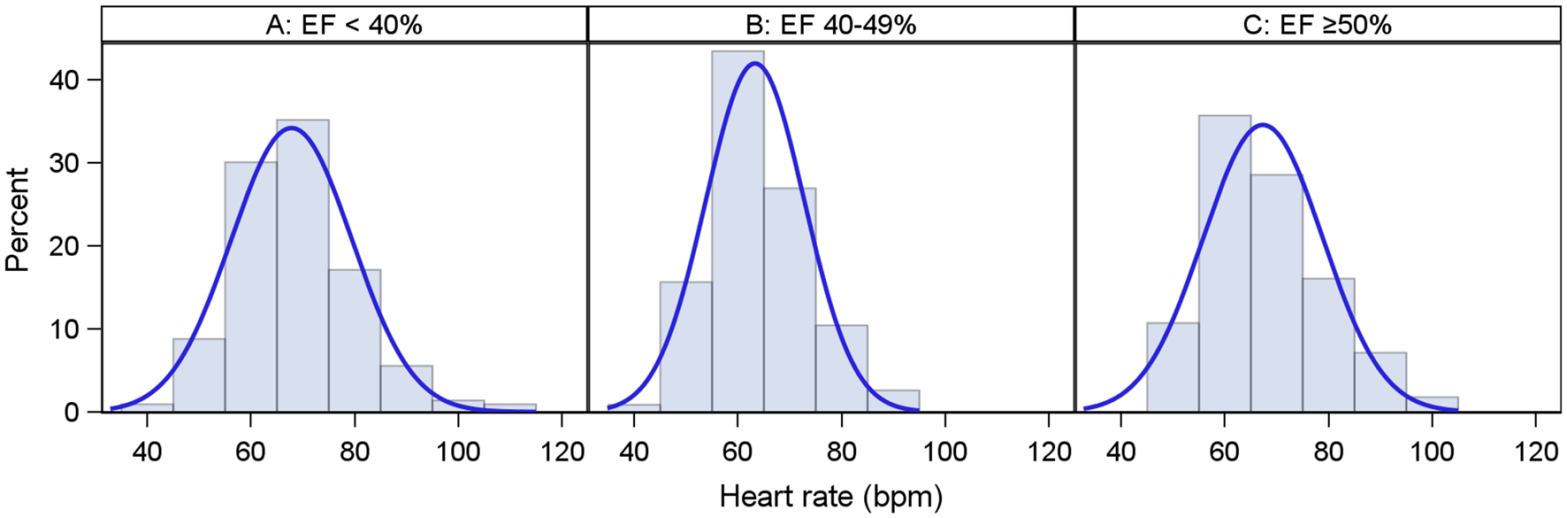
Distribution of heart rate for patients with sinus rhythm and EF <40% (**a**), EF 40–49% (**b**), and EF ≥50% (**c**)

**Fig. 2:**
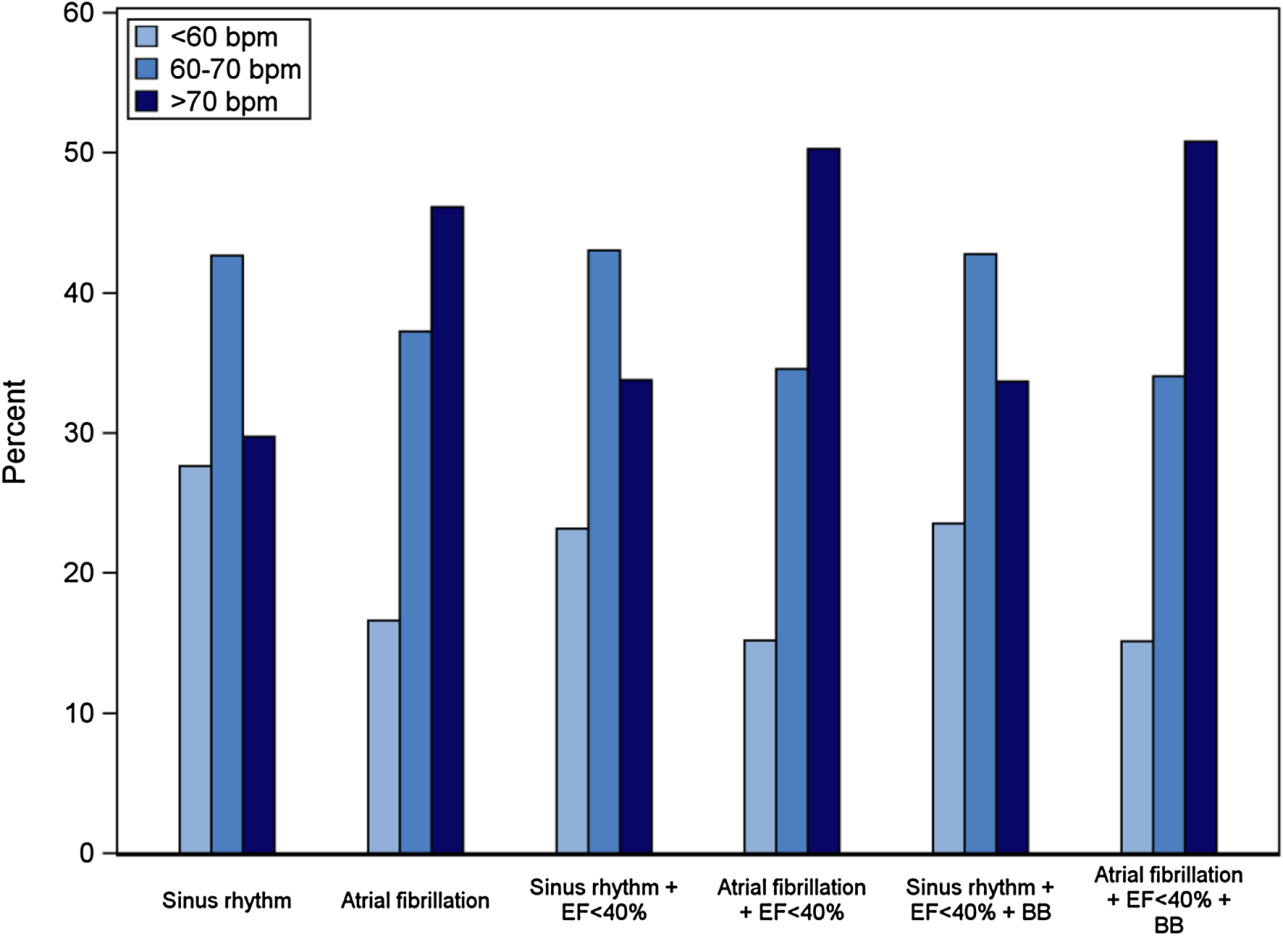
Distribution of heart rate in patients with EF <40%

In the total cohort HR was 68.4 ± 12 bpm with 37.2% of the patients having a HR >70 bpm and 22.2% <60 bpm (Table 2). Patients with HFrEF presented the highest HR (69.8 ± 13 bpm): 41.9% >70 bpm and HFpEF (68.1 ± 12): 33.6% >70 bpm. Patients with HFmrEF had the lowest HR (65.5 ± 11), in which 28.9% had >70 bpm (Table 2; Fig. 1). On average, atrial fibrillation (AF), irrespective of HFrEF, HFmrEF, and HFpEF, had a higher HR and more than 40% of the patients had a HR >70 bpm as compared with sinus rhythm (about 30% of the patients had a HR >70 bpm). A similar pattern was seen in HFrEF in which about 50% of those suffering from AF had a HR >70 bpm, whereas 34% of those with sinus rhythm had a HR >70 bpm. The pattern of HR remained similar between sinus rhythm and AF in HFrEF despite treatment with BBs (Fig. 2).

### Clinical assessment by physician in the overall cohort

Despite that 37% of all HF and 42% of all HFrEF had a HR >70 bpm, 75% of the physicians felt that the patients had optimal HR control, whereas 20% considered the patients to have a HR that was too high.

### HR and influencing factors in HFrEF with sinus rhythm

In HFrEF patients with sinus rhythm 33.6% had a HR >70 bpm. As shown in Tables 3 and 4, when all variables (demographic variables, cardiovascular risk factors, cardiovascular diseases, non-cardiovascular diseases, clinical status, medications, and clinical assessment by physicians) were compared between HR <70 bpm and >70 bpm, only a few of these variables were statistically significant: EF, symptoms of breathlessness and chest pain, and physicians’ clinical assessment, i.e., those HFrEF patients with HR >70 bpm had lower EF, were more symptomatic, and that 49% of the physicians considered a HR >70 bpm optimal, whereas an equal number of physicians felt that a HR >70 bpm was too high (but without further action) (Table 4).

**Table 3:** Comparison of demographics, risk factors, and medical histories for HR ≤70 vs. >70 bpm in all patients with sinus rhythm and EF <40% (HFrEF)

Variable	≤70 bpm (*n* = 143)	>70 bpm (*n* = 73)	*p* value
Age (years)	67.5 (11.9) 68.8 (25.5; 95.3) *n* = 143	63.9 (14.2) 65.8 (19.0; 91.0) *n* = 73	0.068
Male	107 (74.8%)	57 (78.1%)	0.72
LVEF (%)	29.1 (6.4) 30.0 (10.0; 39.0) *n* = 143	25.6 (7.7) 25.0 (10.0; 38.0) *n* = 73	0.0016
NT-pro-BNP (ng/L)	2451 (3429) 1360 (14; 25,600) *n* = 118	3101 (4981) 1239 (81; 27,362) *n* = 66	0.94
Hemoglobin (g/L)	137.4 (16.1) 139.0 (86.0; 175.0) *n* = 123	137.9 (23.7) 140.0 (4.0; 178.0) *n* = 63	0.56
Number of hospitalizations due to heart failure in the past 2 years	0.427 (0.622) 0.000 (0.000; 3.000) *n* = 143	0.616 (0.860) 0.000 (0.000; 3.000) *n* = 73	0.18
Cardiovascular risk factors			
Hypertension	69 (48.3%)	30 (41.1%)	0.39
BMI >30 kg/m2	38 (26.6%)	28 (38.4%)	0.11
Smoking			
Never smoked	62 (43.4%)	27 (37.0%)	
Stopped smoking	58 (40.6%)	36 (49.3%)	
Smoking	23 (16.1%)	10 (13.7%)	0.70
Diabetes	36 (25.2%)	21 (28.8%)	0.68
Alcohol			
Normal consumption	115 (93.5%)	56 (96.6%)	
Previously problematic	5 (4.1%)	2 (3.4%)	
Problematic	3 (2.4%)	0 (0.0%)	0.28
Heredity	32 (22.4%)	26 (36.6%)	0.043
Hypercholesterolemia	59 (41.3%)	22 (30.6%)	0.17
Stress	33 (23.2%)	22 (30.1%)	0.35
Cardiovascular diseases			
Ischemic heart disease	81 (56.6%)	31 (42.5%)	0.067
Primary valvular disease	12 (8.4%)	5 (6.8%)	0.92
Cardiomyopathy	46 (32.2%)	26 (35.6%)	0.72
Myocarditis	3 (2.1%)	1 (1.4%)	1.00
Chronic persistent atrial fibrillation	0 (0.0%)	0 (0.0%)	1.00
Paroxysmal atrial fibrillation	0 (0.0%)	0 (0.0%)	1.00
Cardiac arrest	10 (7.0%)	1 (1.4%)	0.13
VES/VT	28 (19.6%)	14 (19.2%)	1.00
SVT	3 (2.1%)	4 (5.5%)	0.35
Bradycardia	14 (9.8%)	5 (6.8%)	0.65
Non-cardiovascular diseases			
Mild/moderate pulmonary disease	15 (10.5%)	6 (8.2%)	0.79
Severe pulmonary disease	0 (0.0%)	3 (4.1%)	0.075
Asthma	7 (4.9%)	4 (5.5%)	1.00
GFR (cat.)			
GFR <30 ml/min	3 (2.1%)	2 (2.8%)	
GFR 30–60 ml/min	53 (37.3%)	24 (33.8%)	
GFR >60 ml/min	86 (60.6%)	45 (63.4%)	0.79
Missing	1	2	
Stroke without sequelae	10 (7.0%)	2 (2.7%)	0.33
Stroke with sequelae	5 (3.5%)	1 (1.4%)	0.68
Depression	14 (9.8%)	10 (13.7%)	0.52
Impotence	23 (23.5%)	8 (16.7%)	0.47
Malignancy (active)	3 (2.1%)	1 (1.4%)	1.00
Malignancy (stable)	15 (10.5%)	8 (11.0%)	1.00
Malnutrition	1 (0.7%)	2 (2.7%)	0.53
Liver failure	0 (0.0%)	0 (0.0%)	1.00
Thyroid disease	10 (7.0%)	2 (2.7%)	0.33
Gout	21 (14.7%)	5 (6.8%)	0.14
Dementia	0 (0.0%)	1 (1.4%)	0.68
Other important non-cardiovascular disease	13 (9.1%)	8 (11.0%)	0.83
Current status			
Breathlessness—Likert scale			
Never	45 (31.5%)	10 (13.7%)	
Upstairs	75 (52.4%)	51 (69.9%)	
On level ground	20 (14.0%)	7 (9.6%)	
In the shower	3 (2.1%)	2 (2.7%)	
When resting	0 (0.0%)	3 (4.1%)	0.015
Tiredness—Likert scale			
Never	59 (41.3%)	20 (27.4%)	
Upstairs	63 (44.1%)	43 (58.9%)	
On level ground	15 (10.5%)	5 (6.8%)	
In the shower	4 (2.8%)	1 (1.4%)	
When resting	2 (1.4%)	4 (5.5%)	0.12
Chest pain—Likert scale			
Never	128 (89.5%)	71 (97.3%)	
Upstairs	10 (7.0%)	2 (2.7%)	
On level ground	2 (1.4%)	0 (0.0%)	
In the shower	1 (0.7%)	0 (0.0%)	
When resting	2 (1.4%)	0 (0.0%)	0.048
Sitting systolic blood pressure (mmHg)	123.1 (15.8) 120.0 (85.0; 165.0) *n* = 122	123.4 (22.4) 122.0 (54.0; 180.0) *n* = 60	0.94
Standing systolic blood pressure (mmHg)	120.6 (17.6) 120.0 (80.0; 165.0) *n* = 113	121.8 (22.5) 122.5 (70.0; 180.0) *n* = 58	0.80
LBBB	44 (30.8%)	19 (26.0%)	0.57
NYHA			
I	32 (22.4%)	10 (13.7%)	
II	81 (56.6%)	44 (60.3%)	
III	30 (21.0%)	19 (26.0%)	0.14

**Table 4:** Medications and physicians’ opinion regarding a HR ≤70 vs. >70 bpm in patients with sinus rhythm and EF <40%

Variable	≤70 bpm (*n* = 143)	>70 bpm (*n* = 73)	*p* value
Beta-blockers	138 (96.5%)	70 (95.9%)	1.00
Beta-blockers (name)			
Atenolol	1 (0.7%)	0 (0.0%)	
Bisoprolol	53 (37.1%)	31 (42.5%)	
Carvedilol	12 (8.4%)	2 (2.7%)	
Metoprolol	72 (50.3%)	37 (50.7%)	
Not using	5 (3.5%)	3 (4.1%)	0.51
Reasons for not using BBs			
Low blood pressure	0 (0.0%)	1 (1.4%)	0.68
Dizziness	1 (0.7%)	1 (1.4%)	1.00
Raynaud/Claudio	0 (0.0%)	1 (1.4%)	0.68
Pulmonary disease	0 (0.0%)	0 (0.0%)	1.00
Fatigue	0 (0.0%)	2 (2.7%)	0.23
Bradycardia	5 (3.5%)	0 (0.0%)	0.25
Asthma	0 (0.0%)	0 (0.0%)	1.00
Decompensation	0 (0.0%)	0 (0.0%)	1.00
No indication	0 (0.0%)	0 (0.0%)	1.00
Other	1 (0.7%)	1 (1.4%)	1.00
BB dose reached			
≥50 target dose^a^	99 (72.8%)	56 (80.0%)	0.34
Target dose^a^	52 (38.2%)	28 (40.0%)	0.92
>Target dose^a^	2 (1.5%)	0 (0.0%)	0.87
The maximum tolerated dose (physician´s opinion)	129 (93.5%)	59 (84.3%)	0.066
Reasons for not achieving BB target dose			
Low blood pressure	32 (23.2%)	15 (21.4%)	0.92
Fatigue	12 (8.7%)	8 (11.4%)	0.69
Dyspnea	3 (2.2%)	0 (0.0%)	0.58
Dizziness	11 (8.0%)	9 (12.9%)	0.38
Bradycardia	30 (21.7%)	3 (4.3%)	0.0010
Other	14 (10.1%)	10 (14.3%)	0.51
BB tolerated (on treatment with BB)			
No report of side effects	87 (60.8%)	44 (60.3%)	1.00
Nightmares as side effect	5 (3.5%)	2 (2.7%)	1.00
Cold extremities as side effect	16 (11.2%)	3 (4.1%)	0.13
Impotence as side effect	16 (11.2%)	2 (2.7%)	0.049
Depression as side effect	2 (1.4%)	5 (6.8%)	0.090
Tiredness as side effect	26 (18.2%)	17 (23.3%)	0.48
Other side effects	3 (2.1%)	2 (2.7%)	1.00
BB up-titration done at			
Department of Cardiology	110 (80.3%)	59 (83.1%)	
Department of Medicine	22 (16.1%)	11 (15.5%)	
Primary care	5 (3.6%)	1 (1.4%)	0.65
BB duration (years)	3.60 (4.55) 1.50 (0.00; 19.80) *n* = 138	3.01 (4.31) 1.30 (0.00; 18.10) *n* = 69	0.055
RAAS	140 (97.9%)	69 (94.5%)	0.35
ACE inhibitors	92 (64.3%)	44 (60.3%)	0.66
ARB	51 (35.7%)	26 (35.6%)	1.00
ACE inhibitors (name)			
Enalapril	24 (16.8%)	19 (26.0%)	
Lisinopril	0 (0.0%)	1 (1.4%)	
Not using	51 (35.7%)	29 (39.7%)	
Ramipril	68 (47.6%)	24 (32.9%)	0.082
ARB (name)			
Candesartan	38 (26.6%)	18 (24.7%)	
Irbesartan	0 (0.0%)	2 (2.7%)	
Losartan	11 (7.7%)	5 (6.8%)	
Not using A	92 (64.3%)	47 (64.4%)	
Valsartan	2 (1.4%)	1 (1.4%)	0.40
ACE reached the maximum tolerated dose (physician’s opinion)	86 (93.5%)	36 (81.8%)	0.080
ARB reached the maximum tolerated dose (physician’s opinion)	43 (82.7%)	23 (82.1%)	1.00
RAAS reached the maximum tolerated dose (physician’s opinion)	125 (89.3%)	56 (81.2%)	0.16
MRA	84 (58.7%)	42 (57.5%)	0.98
MRA reached the maximum tolerated dose (physician’s opinion)	74 (88.1%)	39 (92.9%)	0.62
Other treatments			
Loop diuretics	79 (55.2%)	40 (54.8%)	1.00
Digitalis	4 (2.8%)	3 (4.1%)	0.88
Statin	94 (65.7%)	42 (57.5%)	0.30
Nitrate	17 (11.9%)	7 (9.6%)	0.79
Other thrombin inhibitors	26 (18.2%)	16 (21.9%)	0.63
ASA	78 (54.5%)	38 (52.1%)	0.84
Anticoagulants	25 (17.5%)	12 (16.4%)	1.00
Antiarrhythmics other than BB	3 (2.1%)	2 (2.7%)	1.00
Ivabradine/procoralan	3 (2.1%)	6 (8.2%)	0.084
Allopur/probenecid	19 (13.3%)	3 (4.1%)	0.051
Device treatments			
Conventional pacemaker	3 (2.1%)	6 (8.2%)	0.084
CRT	20 (14.0%)	9 (12.3%)	0.91
ICD	28 (19.6%)	18 (24.7%)	0.49
Clinical assessment			
Physician considers patient having too low heart rate	4 (2.8%)	1 (1.4%)	0.90
Physician considers patient being optimally treated	129 (90.2%)	36 (49.3%)	<0.0001
Physician considers patient having too high heart rate	10 (7.0%)	36 (49.3%)	<0.0001

^a^ Target dose is calculated only for patients using metoprolol (target = 200 mg), carvedilol (target = 50 mg), and bisoprolol (target = 10 mg)

### Use of BBs in HFrEF with sinus rhythm

As can be seen in Table 4, there were no differences between a HR <70 bpm and a HR >70 bpm in the use of BBs, regardless of prescription, type of BBs, duration of BB use, site for BB up-titration, or dose. In HFrEF with sinus rhythm, BBs were used in 97% (HR <70 bpm) and 96% (HR >70 bpm) in overall population, and in 73% (HR <70 bpm) and 80% (HR >70 bpm) at ≥50% of target dose, 38% (HR <70 bpm) and 40% (HR >70 bpm) at target dose, and 2% (HR <70 bpm) and 0% (HR >70 bpm) at a dose above target dose (Table 4).

### Interaction analysis

**Fig. 3:**
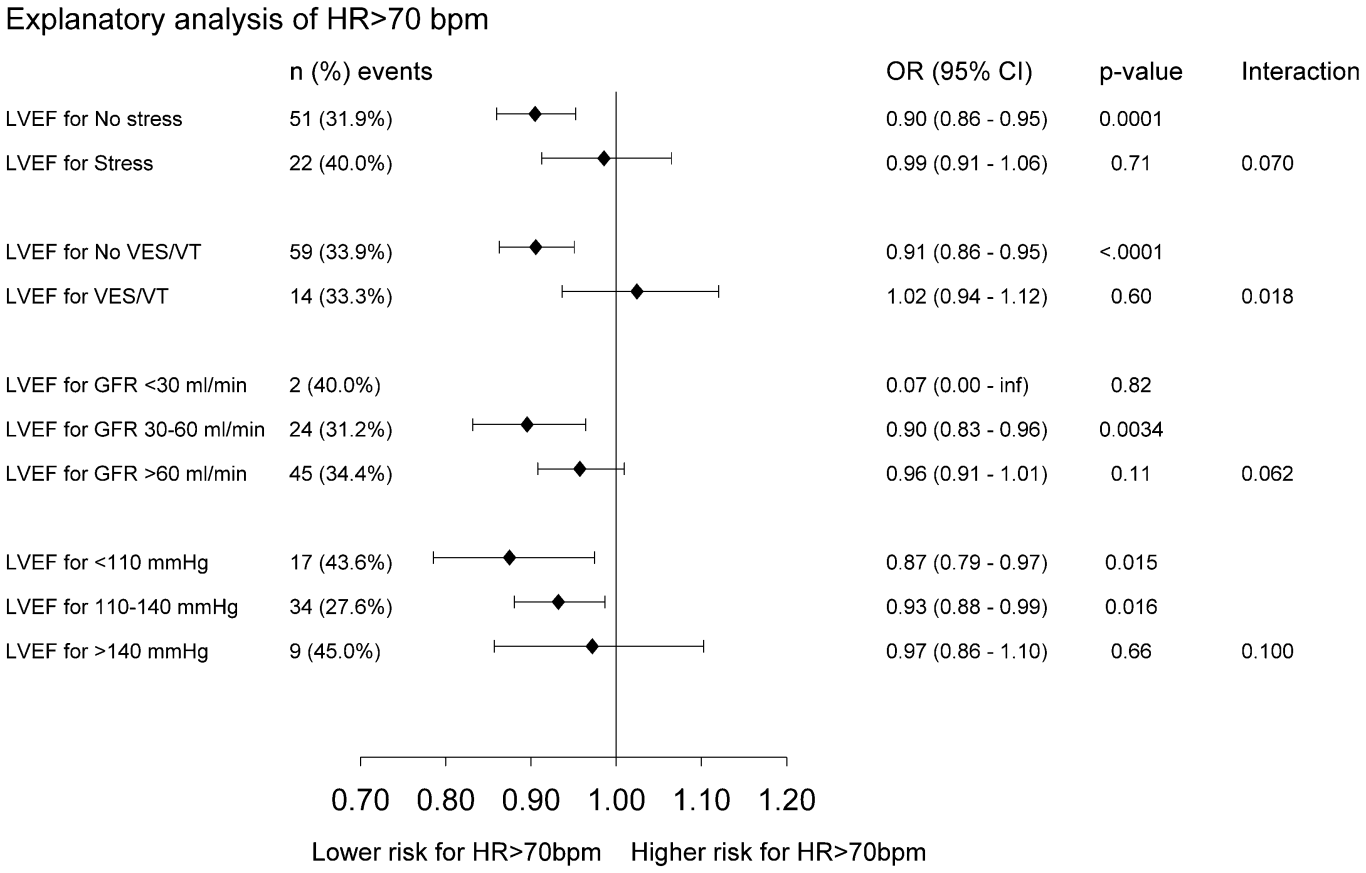
Subgroup analysis of the effect of LVEF on HR in patients with sinus rhythm

Because the current study was aimed to explore possible contributing factors to a HR >70 bpm in HFrEF with sinus rhythm, we analyzed the interaction with EF or BBs leading to the risk of a HR >70 bpm. Low EF is a recognized factor linked to a HR >70 bpm. BBs are assumed to impact HR. Interaction analyses were performed between EF and BBs vs. baseline data that included demographics, medical history, and clinical and laboratory data (Table 5; Fig. 3). There was no significant interaction with BBs but significant interactions between EF and the following variables as explanatory factors of HF >70 bpm were observed: psychological stress, VPC/VT, GFR, and systolic blood pressure. In patients who had no stress, no VPC/VT, lower GFR, and lower SBP (<100 mmHg), EF caused a lower risk for HR >70 bpm, whereas in patients with stress and VPC/VT, higher GFR and higher SBP (>140 mmHg) EF did not affect HR.

**Table 5:** Interaction analyses between LVEF (%) and beta-blockers vs. demographics and clinical and laboratory data in an explanatory analysis of HR ≤70 vs. >70 bpm in all patients with sinus rhythm and EF <40%

Interaction tested with variable	*p* value for interaction with LVEF	*p* value for interaction with BB
Age (years)	0.81	0.55
Sex	0.19	0.96
NT-pro-BNP (ng/L)	0.12	0.57
Hemoglobin (g/L)	0.30	0.89
Number of hospitalizations due to heart failure the past 2 years	0.90	0.83
Hypertension	0.20	1.00
BMI >30 kg/m^2^	0.76	0.95
Smoking	0.21	0.88
Diabetes	0.62	0.95
Heredity	0.15	0.95
Hypercholesterolemia	0.96	0.29
Ischemic heart disease	0.91	0.26
Primary valvular disease	0.69	0.97
Cardiomyopathy	0.38	0.23
Cardiac arrest	0.42	
VES/VT	0.018	
SVT	0.90	
Bradycardia	0.50	0.92
Mild/moderate pulmonary disease	0.49	0.97
Severe pulmonary disease	1.00	
Asthma	0.18	
GFR (cat.)	0.062	0.89
Stroke without sequelae	0.29	0.97
Stroke with sequelae	0.35	
Depression	0.12	0.97
Impotence	0.17	0.95
Malignancy (active)	0.96	
Malignancy (stable)	0.46	0.97
Thyroid disease	0.26	0.98
Sitting systolic blood pressure (mmHg)	0.14	0.76
Sitting systolic blood pressure (cat.)	0.100	0.93
Standing systolic blood pressure (mmHg)	0.37	0.44
Standing systolic blood pressure (cat.)	0.63	0.49
LBBB	0.37	0.97
Chamber pacing	0.45	0.95
NYHA	0.44	0.27
Married/partner	0.62	0.93
Working	0.84	0.95
Retired	0.88	0.95

## Discussions

This study reports suboptimal HR control in stable patients with HFrEF in an outpatient clinical setting. We also report the distribution of HR in different categories of HF: HFrEF, HFmrEF, and HFpEF, both in sinus rhythm and AF, which, to our knowledge, has not been previously reported.

The mean HR of the HFrEF patients in sinus rhythm was 70 bpm with 34% having >70 bpm. This rate was lower than in our previous study (SwedeHF) in which about 47% of the patients had a HR >70 bpm [14]. However, there are several differences: first, the present study was a prospective investigation with a specific aim to study HR and, therefore, ECG was required to register HR at the time of inclusion; in SwedeHF the time point for HR could vary. Second, in the present study all HF patients were stable and in an outpatient clinical setting, whereas most of the patients in SwedeHF were hospitalized. However, the data from our current study were similar to another prospective multicenter study of patients with HFrEF and sinus rhythm in which 32% of the patients had HFs ≥70 bpm [10].

### Possible causes for suboptimal target heart rate in HFrEF and sinus rhythm

Two reasonable questions to ask are: why does HR differ across different studies and why does a HR of >70 bpm still occur in at least one-third of the HFrEF patients? As demonstrated in our study, non-achievement of the recommended target HR was unrelated to age, sex, cardiovascular risk factors, cardiovascular diseases, and comorbidities, but was related to EF and the clinical decision of the responsible physician. From our present and previous study [14], it appears that EF has an important impact on HR (i.e., lower EF is associated with higher HR), possibly implying that left ventricular function is one of the essential driving factors for higher HR.

Clinical assessment by physicians has received increased attention related to their roles in optimizing HF care [10–13], reflecting the awareness of and adherence to guideline-recommended treatment goals. In our study almost half of the physicians regarded a HR >70 bpm as optimal in HFrEF and sinus rhythm though equally many physicians considered a HR >70 bpm as being too high but without any plan for immediate action.

### Role of BBs for suboptimal target HR in HFrEF and sinus rhythm

While the question of how BBs favorably influence the course of HF still remains unanswered, lowering HR is considered very important [18, 19]. Although an increasing number of studies have demonstrated that a substantial proportion of patients with HFrEF does not tolerate the target doses of BBs used in large clinical trials [7, 10, 14, 20], dose issues surrounding BB appear persistent: first, when could we be certain that patients have reached the highest tolerable dose despite being below target dose? Second, how long should dose up-titration continue until it is certain that patients have reached the highest dose tolerable? As long as these questions remain unanswered, the addition of HR-reducing therapies (such as ivabradine) will be postponed or questioned. Moran et al. argued that a lower use of BBs accounted for the difference between those attaining and those not attaining target HRs in stable HFrEF and sinus rhythm [10]. However, these findings could not be confirmed in our study. We did not observe any differences in the use of BBs between patients that had <70 bpm and those that had >70 bpm, nor was there any interaction with BBs in patients with a HR >70 bpm. Both prescription (96%) and achieved target doses (40%) of BBs were higher in our study than in the above-mentioned study (prescription 89% and achieved target doses 25%) [10]. Taken together, these studies seem to suggest that despite differences in the use of BBs, a sizable proportion (approximately one-third) of the patients with HR >70 bpm was similar, suggesting that use of BBs is not the only explanation. Indeed, the proportion of HR >70 bpm is unrelated to the use of BBs as long as the BBs were up-titrated to the highest dose tolerable, which differs individually. As previously shown from the MERIT-HF trial, sicker patients did not tolerate higher doses of BBs, and despite this, the BBs were still effective, suggesting that it is the highest dose tolerable to patients that is all-important [20]. Further, as suggested from a recent meta-analysis, BB efficacy was significant in sinus rhythm, but not in AF, even though both groups showed a reduction in HR [21].

## Limitations

The HF population enrolled in the study may not necessarily reflect the overall HF population. However, similar clinical characteristics in our study as compared with those from SwedeHF suggest the representativeness of our study population. Although participating investigators were encouraged to include patients consecutively we were unable to check that consecutive sampling was conducted.

## Implications

Our data, together with available data [6–10, 14], underline that about one-third of the patients with HFrEF and sinus rhythm did not reach the target HR of <70 bpm as recommended by HF guidelines. However, this cannot be attributed to the use of BBs as long as they are administered in the highest tolerable dose. Further, approximately two-thirds of these patients will not tolerate the target dose, which actually has never been confirmed in a real-world setting.

A possible reason why physicians chose not to add ivabradine when the HR was >70 bpm might be that the recommendations from the EMA and most national pharmaceutical agencies are that ivabradine had an accepted indication if HR is >75 bpm [15–17]. The reason for this discrepancy is that survival benefit was shown in the SHIFT study in a subgroup with a heart rate of 75 bpm or higher [22]. Several observational studies have found an association between elevated HR and poor survival. Our study indicates that among patients with HFrEF, who were in sinus rhythm and on highest tolerable doses of beta-blockers, 14.3% might be eligible for ivabradine, which was similar to a previous study [9].

## Conclusion

In this prospective survey of patients with stable HF in an outpatient clinical setting, we observed suboptimal HR control in HFrEF with sinus rhythm that was unrelated to the use of BBs. Our results support the position that concerted efforts and greater attention to control of HR in patients with HFrEF and sinus rhythm are needed.
